# The efficacy of platelet-rich plasma compared to corticosteroids for partial-thickness rotator cuff tears: a randomized controlled trial

**DOI:** 10.1016/j.jseint.2025.101409

**Published:** 2025-12-02

**Authors:** Mohammad Reza Guity, Mahdi Sahebi, Mohammad Pourfarzaneh, Omid Salkhori, Mahdieh Ghiasi, Nima Bagheri

**Affiliations:** a Joint Reconstruction Research Center, Tehran University of Medical Sciences, Tehran, Iran; b Department of Orthopedic Surgery, Imam Khomeini Hospital Complex, Tehran University of Medical Sciences, Tehran, Iran; c Students' Scientific Research Center (SSRC), Tehran University of Medical Sciences, Tehran, Iran; d Brain and Spinal Cord Injury Research Center, Neuroscience Institute, Tehran University of Medical Sciences, Tehran, Iran

**Keywords:** Rotator cuff tendinopathy, PRP, Corticosteroid, Pain, Functional outcomes, Decision making

## Abstract

**Background:**

Management of partial-thickness rotator cuff tears (PTRCTs) remains debated, especially when conservative treatments fail. Corticosteroid (CS) and platelet-rich plasma (PRP) injections are frequently used, but their comparative effectiveness remains unclear. This study compares PRP and CS injections in improving shoulder range of motion, pain, and patient-reported outcomes in an Iranian population.

**Methods:**

A double-blind, randomized controlled trial was conducted with PTRCT patients receiving ultrasound-guided PRP or CS injections. Assessments included shoulder range of motion, visual analog scale for pain, Simple Shoulder Test, Constant–Murley Score, and Oxford Shoulder Score at baseline, 3 months, and 6 months postinjection.

**Results:**

Out of 208 patients screened, 107 were analyzed (39 PRP, 68 CS). Baseline measures were similar except for external rotation, which was higher in the PRP group (*P* < .05). The CS group showed significantly lower visual analog scale scores at 3 and 6 months (*P* < .001). No significant differences were found in forward flexion, abduction, or internal rotation (*P* > .05). External rotation remained greater in the PRP group throughout (*P* < .001). Simple Shoulder Test and Constant–Murley Scores were significantly higher, and Oxford Shoulder Score significantly lower, in the CS group at both follow-ups (*P* < .001). Both groups showed significant improvement from baseline, except for internal rotation in the PRP group at 6 months (*P* = .248).

**Conclusion:**

Both injections improve clinical outcomes in PTRCT, but CS injections provide superior short- and medium-term pain relief and patient-reported outcomes. CS is preferable for patients prioritizing rapid pain reduction. Further long-term studies are needed to assess sustained effects and safety.

Rotator cuff tendinopathy or tear is a common orthopedic disorder that increases with aging.^13^ This tendinopathy incidence is associated with smoking, hypertension, diabetes, male gender, and high critical shoulder angle.^7^^,^^27^ Rotator cuff tear is categorized into complete and partial-thickness rotator cuff tears (PTRCTs). The frequency of PTRCTs compared to complete tears is reported to be higher, and noticeably, most complete tears are attributed to partial tears.^16^ PTRCTs are among the most common shoulder injuries, with a prevalence of approximately 4% at age <40 years, 26% at age >60 years, and 20% in overall asymptomatic individuals.^8^^,^^14^ Surgical management of full-thickness tears is associated with generally good results^20^; however, there is no consensus regarding treating PTRCTs that fail to respond to activity modification and physical therapy. Therefore, the efficacy of conservative treatments remains a matter of debate.^8^ Conservative management, which includes analgesic drugs such as oral nonsteroidal anti-inflammatory drugs, physical therapy, and various injection types, is regarded as the first-line treatment for PTRCT.^14^ In contrast, surgical intervention is typically recommended for patients who do not respond to conservative treatment after 3 to six months and for younger individuals with traumatic tears.^17^ Options for injection therapy consist of corticosteroids (CSs), platelet-rich plasma (PRP), hyaluronic acid, and botulinum toxin.^1^^,^^15^ In recent years, PRP injection therapies have shown great potential for rotator cuff tendinopathy problems as well as other tendon- and joint-related disorders.^3^^,^^24^ PRP injection stimulates natural healing through the growth factors found in platelets. It accelerates the physiological healing process, supports cell connection, reduces pain, and has anti-inflammatory and antibacterial properties.^21^^,^^22^ Several studies have compared PRP to CS injection in treating rotator cuff lesions; however, the question of which method is more effective remains unresolved.^2^^,^^12^^,^^22^^,^^23^ Few studies have assessed the efficacy of PRP vs. CSs in the Iranian population. This study's primary aim is to evaluate further the effectiveness of PRP vs. CSs in improving shoulder range of motion (ROM) and alleviating pain by assessing visual analog scale (VAS) scores at 3-month and 6-month follow-ups. The secondary aim is to compare the efficacy of these 2 injections in improving patient-reported outcome measures, including the Simple Shoulder Test (SST), Constant–Murley Score (CMS), Oxford Shoulder Score (OSS), and abduction force in this population.

## Materials and methods

### Study design and ethical approval

A double-blind randomized controlled trial (RCT) study was conducted on patients diagnosed with PTRCT in the orthopedic clinic of Imam Khomeini Hospital Complex, a nationwide referral hospital in Tehran, Iran, affiliated with the Tehran University of Medical Sciences. The institutional review board of the Tehran University of Medical Sciences (IR.TUMS.IKHC.REC.1403.028) obtained ethical approval before enrolling the patients, and all the participants received written consent. The study's aims, confidentiality, and freedom of participation were explained to all patients before enrolling. This trial was registered in Iranian registry of clinical trials (irct.behdasht.gov.ir, registration code: IRCT20230608058415N1).

### Enrollment of participants and sample size calculation

The inclusion criteria were as follows: (a) aged between 30 and 70 years, (b) both sexes, (c) PTRCT diagnosis with clinical signs (eg, pain in the shoulder and restriction of active motion), (d) magnetic resonance imaging confirmation of PTRCT, (e) no contraindications for shoulder injection, and (f) the ability to give informed consent. The patients were excluded if they had: (a) previous history of shoulder injection or shoulder surgery, (b) frozen shoulder diagnosis, (c) shoulder girdle fractures, (d) complete rotator cuff tear or other pathologies of shoulder (glenohumeral arthrosis, calcific tendinitis, shoulder instability, and acromioclavicular joint pathology), (e) active tumor or hematological malignancies, (f) coagulation disorders or anticoagulant use, or (g) infection.

The minimum requisite sample size for the 2 groups was determined using the G∗Power 3.1.9.2 software (Heinrich Heine Universität, Düsseldorf, Germany) before participant enrollment. Based on a calculated effect size of 0.8, an α level of 0.05, a power of 80%, and an allocation ratio of 1:2, the a priori power analysis determined a total sample size of at least 60 participants. The participants were randomized to receive PRP or CS (methylprednisolone) injection (20 patients in the PRP group and 40 in the CS group). The randomization was conducted using a computer-generated list, and the allocation was concealed within a series of numbered envelopes. The orthopedic specialist who administered the injection, the patient, and the follow-up examiner were not aware of which injection had been administered.

### Platelet-rich plasma preparation

A total of 40 milliliters of venous blood was drawn from participants. This sample was mixed with 6 milliliters of anticoagulant and distributed equally into 4 tubes, which were then centrifuged for 15 minutes at a speed of 1800 rpm. Next, the resulting plasma was transferred to a separate tube, followed by a second round of centrifugation at the same speed of 1800 rpm for 12 minutes. Ultimately, 6 milliliters of platelet-rich liquid, concentrated 5-6 times, was prepared. To maintain study blindness, the CS group also had blood samples taken before the injection to check their complete blood count.

### Platelet-rich plasma and corticosteroid injection

An orthopedic specialist performed all injections with the aid of an ultrasound guide. In the PRP group, the 6-milliliter plasma liquid was administered. In the other group, 1 ampoule of methylprednisolone with a dose of 40 mg/ml was administered as a 1-milliliter solution. The injection procedure was performed using a posterolateral approach, positioned approximately 1.5 fingerbreadths below the posterolateral corner of the acromion, without the use of local anesthesia. The needle was adeptly maneuvered along the superior border of the rotator cuff into the subacromial space. If the needle tip came into contact with the undersurface of the acromion, slight withdrawal of the needle facilitated the smooth administration of the content.

### Clinical assessment

The clinical parameters, which included shoulder ROM, the VAS, SST, CMS, and OSS, were evaluated at baseline, 3 months, and 6 months following PRP or CS injection. Shoulder ROM was assessed when the patient was seated. A goniometer measured how much the patient could flex or abduct the shoulder. External rotation and internal rotation of the shoulders were assessed with the patient's arm in a resting position and at a 45° flexion position, respectively. Muscle power for shoulder abduction was evaluated by measuring the maximal isometric contraction of the abductor muscles at baseline and 3 months after injection. This evaluation was performed using an isokinetic handheld dynamometer with the shoulder positioned at 45° abduction, the elbow at 90° flexion, and the arm internally rotated without torso stabilization. The VAS is a pain measurement scale, where 0 indicates the absence of pain and 10 signifies unbearable pain.^6^ The SST consists of 12 shoulder-specific questions to which the patient answers "yes" or "no." The questions ask about strength, function, and ROM. Zero is considered the worst score, with 12 being the best score.^9^ The CMS is a standardized scale used to evaluate shoulder function, with a maximum score of 100 indicating optimal shoulder performance. It has been utilized in numerous studies to assess shoulder-related outcomes.^10^^,^^18^ OSS contains 12 items, each with 5 answers, starting with 1 (best/fewest symptoms) to 5 (worst/most severe), which is awarded to correspond to the patient's symptoms. The total score yields a maximum of 60, with higher scores indicating greater disability.^26^

### Statistical analysis

Statistical analyses were performed using Statistical Package for the Social Sciences version 27.0 (IBM Corp, Armonk, NY, USA). Continuous variables were expressed as mean ± standard deviation or median (interquartile range) as appropriate. Categorical data were expressed as a percentage and frequency. Continuous numeric variables were assessed for the normality of their distribution using the Kolmogorov-Smirnov test. Normally distributed measurement data are presented as the mean ± standard deviation, while between-group comparisons were made using Student *t* tests. Non-normally distributed data are presented as the median (interquartile range) and statistically analyzed using Mann–Whitney *U* (for independent samples) or Wilcoxon signed-rank (for dependent samples) tests. The significance level was set at *P* < .05 for all analyses.

## Results

### General characteristics

**Figure 1:**
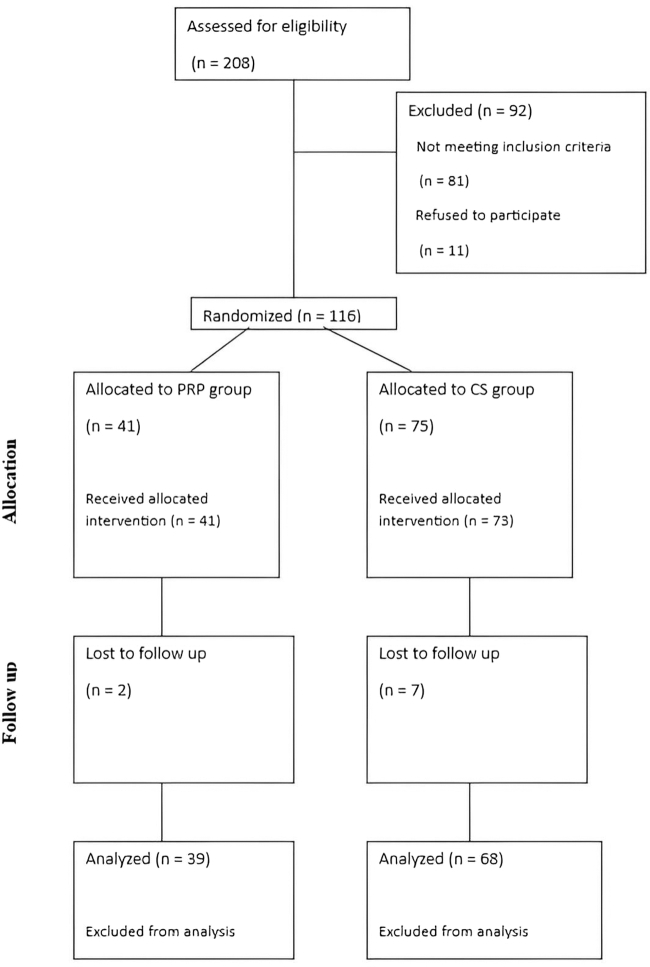
CONSORT flow diagram showing participants' inclusion, exclusion, and grouping. *CONSORT*, Consolidated Standards of Reporting Trials; *CS*, corticosteroid; *PRP*, platelet-rich plasma.

**Figure 2:**
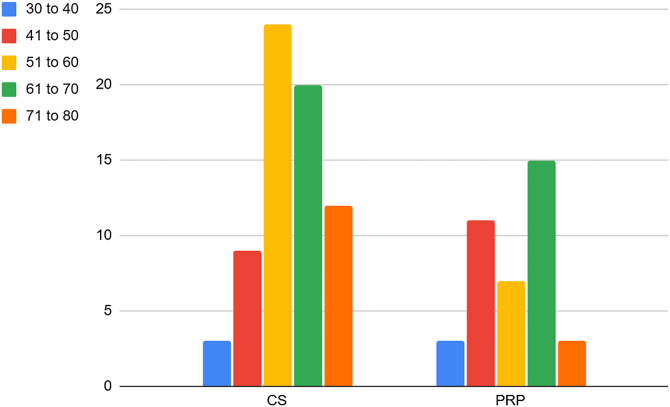
Age distribution across 2 groups (by years). *CS*, corticosteroid; *PRP*, platelet-rich plasma.

Among the 208 individuals who visited our clinic between June and September 2024, 127 patients were eligible for enrollment in the study. Of these, 116 provided informed consent and were randomized into groups at an allocation ratio 1:2 (41 in the PRP group and 75 in the CS group). One hundred seven patients were included in the analysis (39 in the PRP group and 68 in the CS group). Fig. 1 illustrates the Consolidated Standards of Reporting Trials flow diagram of patient inclusion and grouping. The median age in both groups was 60, ranging from 55 to 70 in the CS group (50% female) and 50 to 67 in the PRP group (54% female). The age group distribution of patients across 2 groups is depicted in Fig. 2. The demographic baseline characteristics are shown in Table I.

**Table I:** Baseline demographic characteristics of participants.

Groups parameters	PRP group N = 39	CS group N = 68
Age (yr)	60 (50-67)	60 (55-70)
Male/female	18/21	34/34
Hand dominancy (left/right)	6/33	18/50
Affected shoulder (left/right)	17/22	41/27
Forward flexion (◦)	112.82 ± 31.61	111.03 ± 37.02
Abduction (◦)	106.92 ± 30.18	111.62 ± 26.68
Abduction force (N)	3.53 ± 0.85	3.54 ± 0.80
Internal rotation (◦)	99.49 ± 27.67	98.53 ± 22.01
External rotation (◦)	56.79 ± 17.11	39.71 ± 13.71
VAS	7.10 ± 1.93	6.87 ± 0.99
SST	5.69 ± 2.48	5.75 ± 1.84
CMS	44.77 ± 10.23	45.88 ± 11.98
OSS	37.49 ± 8.89	39.97 ± 8.29

*CS*, corticosteroid; *PRP*, platelet-rich plasma; *VAS*, visual analog scale; *SST*, Simple Shoulder Test; *CMS*, Constant–Murley Score; *OSS*, Oxford Shoulder Score.

### Primary outcomes

**Figure 3:**
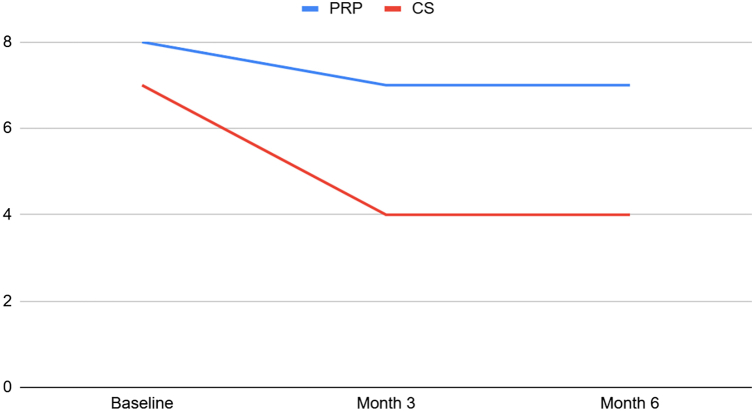
VAS scores in 2 groups at baseline, 3-, and 6-month follow-ups. *VAS*, visual analog scale; *CS*, corticosteroid; *PRP*, platelet-rich plasma.

The VAS score (Fig. 3) was significantly lower in the CS group at 3- and 6-month follow-ups (*P* < .001). There was no statistically significant difference regarding the forward flexion, abduction, and internal rotation range at 3- and 6-month follow-ups (*P* > .05). The external rotation range was statistically higher in the PRP group at baseline, 3-, and 6-month follow-ups (*P* < .001). There was no statistically significant difference at baseline when comparing the 2 groups among other parameters (*P* > .05) (Table II). Similar patterns were also observed in the subgroup analysis of the primary outcome across 3 major age groups (41-50 years, 51-60 years, and 61-70 years), which encompass most patients, especially for VAS (Table III). In addition, at 3- and 6-month follow-ups, all of the primary outcomes were compared to their baseline; all of the parameters exhibited statistically significant differences in both groups (*P* < .05) except internal rotation of the PRP group at 6-month follow-up (*P* = .248).

**Table II:** Primary outcomes.

Outcome	Baseline	Month 3	Month 6
VAS			
PRP	8 (5-9)	7 (5-8)	7 (6-8)
CS	7 (6-8)	4 (3.25-5)	4 (3-5)
*P* value	.165	<.001	<.001
Forward flexion (◦)			
PRP	120 (90-130)	120 (90-150)	120 (90-150)
CS	120 (90-140)	130 (100-150)	130 (110-150)
*P* value	.904	.889	.490
Abduction (◦)			
PRP	100 (90-120)	120 (90-150)	120 (100-150)
CS	110 (90-130)	120 (100-137.5)	130 (110-150)
*P* value	.498	.764	.125
Internal rotation (◦)			
PRP	105 (75-120)	120 (90-120)	110 (90-120)
CS	100 (90-117.5)	110 (100-127.5)	110 (100-120)
*P* value	.572	.606	.275
External rotation (◦)			
PRP	60 (50-70)	60 (60-70)	60 (50-70)
CS	40 (30-50)	50 (40-50)	50 (40-60)
*P* value	<.001	<.001	<.001

*CS*, corticosteroid; *PRP*, platelet-rich plasma; *VAS*, visual analog scale.

**Table III:** Primary outcomes sub-group analysis by years.

Outcome	Baseline			Month 3			Month 6		
	PRP	CS	*P* value	PRP	CS	*P* value	PRP	CS	*P* value
VAS									
41-50	8 (6-9)	6 (6-7)	.230	7 (6-8)	4 (3.5-5)	.003	7 (6-8)	4 (2.5-5)	.002
51-60	8 (5-8)	7 (6-7)	.627	7 (5-8)	4 (3-5)	<.001	7 (5-8)	3.5 (3-4.75)	<.001
61-70	7 (5-9)	7 (6.25-8)	.831	7 (5-7)	4 (3.25-4.75)	<.001	7 (5-7)	4 (3-4)	<.001
Forward flexion (◦)									
41-50	120 (90-150)	120 (85-130)	.656	120 (100-160)	140 (105-160)	.824	120 (100-160)	140 (110-165)	.603
51-60	120 (60-130)	100 (50-137.5)	.764	120 (90-150)	110 (70-157.5)	.800	120 (90-150)	115 (80-157.5)	1.000
61-70	120 (100-130)	130 (95-140)	.268	120 (110-140)	130 (102.5-157.5)	.458	120 (100-140)	130 (112.5-157.5)	.240
Abduction (◦)									
41-50	120 (90-130)	100 (90-160)	.603	140 (90-150)	150 (90-150)	.766	140 (100-150)	150 (110-160)	.331
51-60	120 (60-120)	110 (90-130)	.317	120 (90-160)	120 (100-130)	.945	120 (90-160)	130 (105-150)	.444
61-70	100 (90-130)	120 (90-120)	.934	120 (100-150)	120 (100-127.5)	.681	120 (100-150)	105 (130-140)	.521
Internal rotation (◦)									
41-50	105 (75-120)	100 (75-120)	.824	105 (75-120)	100 (85-130)	.824	100 (75-120)	110 (90-130)	.603
51-60	90 (75-105)	90 (72.5-117.5)	.729	110 (90-120)	105 (92.5-120)	.945	110 (90-120)	110 (100-120)	1.000
61-70	105 (75-120)	100 (90-107.5)	.542	120 (70-120)	105 (100-127.5)	.755	110 (80-120)	110 (100-127.5)	.458
External rotation (◦)									
41-50	60 (50-70)	30 (30-55)	.080	70 (60-70)	50 (35-65)	.131	70 (60-70)	60 (40-65)	.201
51-60	60 (30-70)	40 (30-50)	.068	70 (50-70)	40 (40-50)	.022	70 (45-70)	45 (40-50)	.085
61-70	60 (50-70)	40 (30-50)	.002	60 (50-70)	45 (40-60)	.013	60 (50-70)	50 (40-60)	.025

*CS*, corticosteroid; *PRP*, platelet-rich plasma; *VAS*, visual analog scale.

### Secondary outcomes

**Figure 4:**
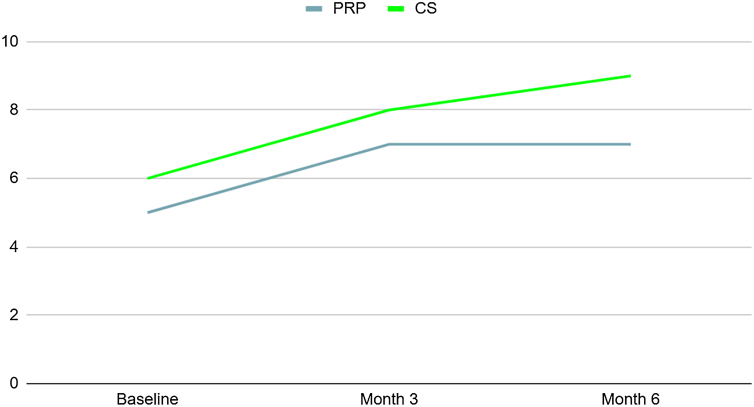
SST scores in 2 groups at baseline, 3-, and 6-month follow-ups. *SST*, Simple Shoulder Test; *CS*, corticosteroid; *PRP*, platelet-rich plasma.

**Figure 5:**
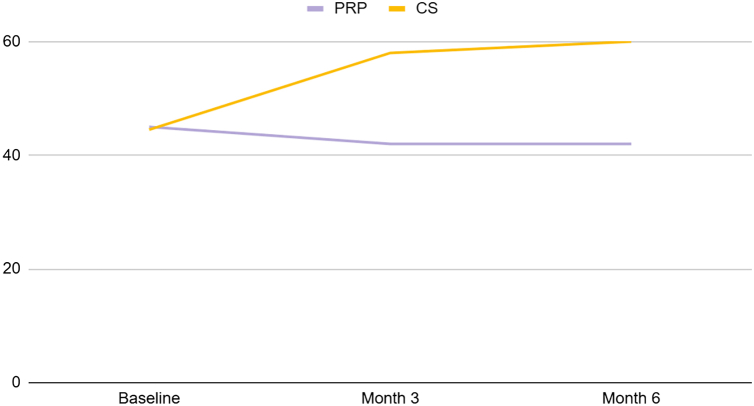
CMS cores in 2 groups at baseline, 3-, and 6-month follow-ups. *CMS*, Constant–Murley Score; CS, corticosteroid; *PRP*, platelet-rich plasma.

The SST and CMS (Figs. 4 and 5) were statistically higher in the CS group at 3- and 6-month follow-ups (*P* < .05), and the OSS was statistically lower in the CS group at 3- and 6-month follow-ups (*P* < .05). There was no statistically significant difference regarding the abduction force at 3-month follow-up (*P* > .05). There was no statistically significant difference at baseline between the 2 groups across all parameters (*P* > .05) (Table IV). Furthermore, at the 3-month and 6-month follow-ups, all secondary outcomes were compared to their baseline, demonstrating statistically significant differences in both groups (*P* < .05). The details of secondary outcome subgroup analysis across 3 major age groups are depicted in Table V.

**Table IV:** Secondary outcomes.

Outcome	Baseline	Month 3	Month 6
SST			
PRP	5 (4-7)	7 (4-9)	7 (5-8)
CS	6 (5-7)	8 (7-10)	9 (7-10)
*P* value	.449	.003	<.001
CMS			
PRP	45 (36-54)	42 (40-50)	42 (39-48)
CS	44.50 (38-53.75)	58 (49.25-69.75)	60 (50-70.75)
*P* value	.688	<.001	<.001
OSS			
PRP	38 (31-45)	36 (28-41)	38 (30-44)
CS	40 (34-46.75)	21.50 (14.25-32)	22 (15-31)
*P* value	.300	<.001	<.001
Abduction force (N)			
PRP	4 (3-4)	4 (3-4)	
CS	4 (3-4)	4 (3-4)	
*P* value	.934	.667	

*CS*, corticosteroid; *PRP*, platelet-rich plasma; *SST*, Simple Shoulder Test; *CMS*, Constant–Murley Score; *OSS*, Oxford Shoulder Score.

**Table V:** Secondary outcomes sub-group analysis by years.

Outcome	Baseline			Month 3			Month 6		
	PRP	CS	*P* value	PRP	CS	*P* value	PRP	CS	*P* value
SST									
41-50	5 (3-8)	6 (3.5-8)	.710	5 (4-10)	9 (6.5-11)	.295	6 (4-10)	9 (7-11)	.175
51-60	5 (4-5)	5 (4-7)	.341	7 (3-10)	7 (6-9)	.595	7 (4-8)	7 (7-9)	.216
61-70	6 (4-8)	6 (4.25-7)	.831	7 (5-9)	8.5 (7.25-10)	.114	8 (5-9)	8 (7.25-10)	.099
CMS									
41-50	45 (36-54)	41 (38.5-53.5)	.941	42 (40-52)	52 (48-65)	.046	40 (35-50)	55 (49.5-66.5)	.006
51-60	36 (33-39)	42 (31.25-53)	.532	40 (39-42)	54 (45-69.75)	<.001	40 (38-42)	58.5 (44-73.25)	<.001
61-70	45 (35-56)	44.5 (38.75-51.75)	.856	46 (41-57)	59 (50.5-68)	.016	45 (40-55)	60.5 (51-69.5)	.006
OSS									
41-50	38 (34-44)	36 (30-49)	1.000	38 (26-41)	12 (11-34)	.01	38 (32-45)	13 (11.5-35)	.012
51-60	40 (31-48)	42 (37.25-48)	.532	40 (27-48)	26.5 (17.25-37.25)	.038	39 (30-48)	26 (18.25-34.5)	.017
61-70	34 (26-46)	41 (32.75-46)	.227	34 (27-40)	22 (15-30.5)	.003	36 (30-40)	22.5 (15-29.75)	<.001
Abduction force (N)									
41-50	3 (3-4)	4 (3-4)	.456	4 (3-4)	4 (3.5-5)	.175			
51-60	4 (3-4)	4 (3-4)	.800	4 (3-4)	4 (3-4)	.835			
61-70	4 (3-4)	4 (3-4)	.521	4 (3-4)	4 (3-4.75)	.681			

*CS*, corticosteroid; *PRP*, platelet-rich plasma; *SST*, Simple Shoulder Test; *CMS*, Constant–Murley Score; *OSS*, Oxford Shoulder Score.

## Discussion

To the best of the authors' knowledge, this is the first study that compares the outcomes of PRP vs. CS injection at a 6-month follow-up in the Iranian population. The principal finding of this study is that both interventions are effective methods for managing PTRCTs; however, in terms of subjective pain assessment, the CS group exhibited a lower VAS score at the 3- and 6-month follow-ups.

Regarding shoulder ROM, there was no statistically significant difference between the 2 groups except for external rotation, in which the PRP group demonstrated better function. Although due to baseline discrepancies between the 2 groups, we could not determine whether the PRP group's external rotation ROM superiority in follow-ups was due to the type of injection. We also evaluated 2 groups based on 3 established functional patient-reported outcome measure scores (SST, CMS, and OSS), all of which indicated a better result for CS group at 3- and 6-month follow-ups.

Dadgostar et al^5^ conducted the first RCT on this issue in the Iranian population with a 3-month follow-up. They found that PRP produces similar results to CS in most clinical aspects among patients (including the Western Ontario Rotator Cuff and Disabilities of the Arm, Shoulder, and Hand [DASH] scores) with rotator cuff tendinopathies; however, pain (VAS) and ROM may significantly improve with PRP. Another RCT by Shams et al^23^ compared the effects of PRP to those of CS injections in patients with PTRCT. Both injection groups demonstrated statistically significant improvements in clinical outcomes over time compared to their preinjection status. A statistically significant difference was observed between the PRP group and the CS group 12 weeks after injection regarding VAS, the American Shoulder and Elbow Surgeons Standardized Shoulder Assessment Form, CMS, and SST, favoring the PRP group; however, statistically significant disparities could not be seen after 6 months.

In an RCT by Barreto et al,^2^ the effectiveness of PRP in treating patients with rotator cuff syndrome was compared to therapy with subacromial CS injections at baseline, 1 month, three months, and six months. No significant differences were found when comparing the results of the DASH score, the University of California - Los Angles Shoulder Rating Scale, and the CMS of the 2 groups at baseline and after 1, 3, and six months of the subacromial injection. After the treatment, both groups showed a significant improvement in the DASH and University of California - Los Angles scores compared to the baseline. However, the CMS for the steroid group six months after treatment was lower than the baseline. Annaniemi et al^1^ conducted an 18-month follow-up retrospective study to compare PRP and CS for rotator cuff tendinopathy. Both treatments improved patient symptoms, but neither resulted in a significantly better Western Ontario Rotator Cuff, VAS, or ROM result. The general outcomes of these studies are in accordance with our findings; however, there are some discrepancies regarding the superiority of pain improvement by these interventions. Our findings indicate that CS injections provide superior short- and medium-term pain relief compared to PRP in this patient population. These differences could be attributed to various PRP preparation protocols, the diversity of injection approaches, the method of patient selection, and possible variation in effect between sites of tendinopathy.

In addition, similar results have been reported for calcific tendinitis. Oudelaar et al^19^ conducted a double blind RCT to evaluate the efficacy of adjuvant application of PRP vs. CS after needle aspiration of calcific deposits of rotator cuff calcific tendinitis. At the 6-week follow-up, a clinically relevant difference in favor of CS was found for all scores (CMS, DASH, OSS, EuroQol-5D) except for the numeric rating scale for pain, and clinically relevant differences in favor of PRP were only seen at the 6-month follow-up for numeric rating scale and CMS scores. At the 1- and 2-year follow-ups, the results between the groups were comparable.

This study has several limitations. Firstly, the follow-up period was limited to six months, and longer-term outcomes, including structural tendon healing, were not analyzed. Although CS application has been recognized as a known beneficial method of tendinopathies' short-term management and has indicated clinically significant outcomes in the literature, it may come with side effects in long-term follow-ups when compared to other injectable materials.^4^^,^^11^ Another aspect of this matter is that CS injections tend to be ineffective when administered several weeks after the onset of the pathology,^25^ which could not be evaluated in our study design. Since this was a clinical trial, some participants missed their follow-up appointments and were lost to follow-up during the study. The absence of follow-up imaging data, such as magnetic resonance imaging or ultrasound, which offer valuable insights into tendon integrity and healing—particularly in assessing the regenerative potential of PRP—was another limitation of this study. Also, variability in PRP preparation protocols could impact reproducibility and generalizability. Furthermore, the sample size, although adequate for primary outcomes, may not be sufficient to detect rare adverse events or permit extensive subgroup analyses.

## Conclusion

This RCT demonstrates that both PRP and CS lead to significant improvements in most clinical aspects for patients with PTRCTs. CS injections offer better pain relief and functional improvement compared to PRP over a 6-month follow-up, suggesting that the overall effectiveness of PRP may be limited in this context. Clinicians should consider the benefits and risks of each intervention, taking into account patient preferences, comorbidities, and functional demands. Further research with longer follow-up and consecutive imaging is needed to clarify the optimal role of PRP in managing rotator cuff tendinopathies.

## Disclaimers:

Funding: No funding was disclosed by the authors.

Conflicts of interest: The authors, their immediate families, and any research foundation with which they are affiliated have not received any financial payments or other benefits from any commercial entity related to the subject of this article.
